# A Mathematical Model for Eph/Ephrin-Directed Segregation of Intermingled Cells

**DOI:** 10.1371/journal.pone.0111803

**Published:** 2014-12-01

**Authors:** Rotem Aharon, Peter W. Janes, Anthony W. Burgess, Kais Hamza, Fima Klebaner, Martin Lackmann

**Affiliations:** 1 School of Mathematical Sciences, Monash University, Clayton, Victoria, Australia; 2 Department of Biochemistry & Molecular Biology, Monash University, Clayton, Victoria, Australia; 3 Structural Biology Division, The Walter and Eliza Hall Institute, Parkville, Victoria, Australia; University of Parma, Italy

## Abstract

Eph receptors, the largest family of receptor tyrosine kinases, control cell-cell adhesion/de-adhesion, cell morphology and cell positioning through interaction with cell surface ephrin ligands. Bi-directional signalling from the Eph and ephrin complexes on interacting cells have a significant role in controlling normal tissue development and oncogenic tissue patterning. Eph-mediated tissue patterning is based on the fine-tuned balance of adhesion and de-adhesion reactions between distinct Eph- and ephrin-expressing cell populations, and adhesion within like populations (expressing either Eph or ephrin). Here we develop a stochastic, Lagrangian model that is based on Eph/ephrin biology: incorporating independent Brownian motion to describe cell movement and a deterministic term (the drift term) to represent repulsive and adhesive interactions between neighbouring cells. Comparison between the experimental and computer simulated Eph/ephrin cell patterning events shows that the model recapitulates the dynamics of cell-cell segregation and cell cluster formation. Moreover, by modulating the term for Eph/ephrin-mediated repulsion, the model can be tuned to match the actual behaviour of cells with different levels of Eph expression or activity. Together the results of our experiments and modelling suggest that the complexity of Eph/ephrin signalling mechanisms that control cell-cell interactions can be described well by a mathematical model with a single term balancing adhesion and de-adhesion between interacting cells. This model allows reliable prediction of Eph/ephrin-dependent control of cell patterning behaviour.

## Introduction

Eph receptors (Ephs) are the largest subfamily of receptor tyrosine kinases (RTKs) [1]. The Eph cell-cell contact dependent interaction with cell-bound ephrin ligands orchestrates cell positioning, tissue and organ patterning and controls cell survival during normal and neoplastic development [1]–[4]. In humans, five GPI cell surface-bound type-A ephrins and three transmembrane type-B ephrins interact with nine EphA and five EphB family members, respectively, initiating receptor clustering, tyrosine phosphorylation and downstream forward signalling into Eph-bearing cells [1], [5], [6]. Concurrently, ligated ephrins are drawn into a signalling cluster on the opposing cell surface [7], so that mutually dependent responses to Eph/ephrin interactions are relayed into both cell populations. As a direct consequence of Eph/ephrin signalling, cells undergo changes in the configuration of their actin cytoskeleton and morphology [8]–[10], their contact to neighbouring cells, substrate adhesion, motility and their viability [11]–[14], with downstream effects on cell invasion, tissue boundary formation and on specialised secretory or immune functions [1], [5], [15].

Eph/ephrin mediated tissue patterning has been modelled experimentally using isolated zebrafish embryo caps [16] and cultured epithelial cell lines [17]–[19]. Co-culture of cells expressing either an Eph receptor or corresponding ephrin binding partner(s) can lead either to adhesion and intermingling, or de-adhesion and cell-cell segregation, forming boundaries between the two cell populations. Contrary to chemotactic proteins, cell-cell contact-dependent Eph/ephrin signalling does not direct the collective migration of responsive cell populations, but controls the position of individual cells in relation to their direct neighbours [1], [5], [6]. During developmental patterning, overlapping expression gradients of multiple Ephs and ephrins [20], [21], together with integrated signals from all of the cell surface Eph receptors that compete for available ephrin targets, determine the final position and interaction partners for migrating cells [22]. The complexity of the Eph/ephrin clusters and the associated signalling pathways is only beginning to be appreciated, but it is clear that the net outcomes depend on cell surface concentration, kinase-signalling capacity, type of co-expressed Ephs and ephrins and crosstalk with other signaling systems [5]. It is likely that the difficulties of interpreting the consequences of Eph signalling at cellular, organ and whole organism levels is (at least partially) responsible for the confusing and often contradictory literature [1], [5], [6].

There is an increasing appreciation that the complexity of the Eph signalling network, which relays the input from a large number of parallel cues into a range of fine-tuned cellular responses, requires mathematical modeling to reliably predict the signalling outcomes resulting from Eph/ephrin interaction [7], [17], [22]–[24]. Current mathematical and computational models describing cell movement have been based on different aspects of cell motility, including cytoplasm dynamics [25], the growth of actin filaments [26], and the distribution of adhesion proteins in the cell membrane [27]. More recently, integrated models of several aspects of cell movement have been reported [28]–[30]. However, when considering migration distances that greatly exceed the length of a single cell, and migration times that are much longer than the cell's ‘persistence’ time, the Mean Square Displacement (MSD) of a single cell can be regarded as linear in time [27]. It is thus sufficient to represent the motion of a single cell by a random walk in the discreet case, or by a Brownian motion in the continuous case [31]. Accordingly, for the modeling of cell populations where the motion of a large number of cells is averaged, diffusion equations can be derived and can be used to describe cell chemotaxis [32], cell migration on growing domains [33], or cancerous cell invasion [34].

Models for cell-cell interactions within large cell populations commonly adopt an ‘agent-based’ approach which assumes each ‘agent’ (cell) follows a set of rules governing a random walk exclusion process (where each cell moves randomly on a grid but cannot move to a site already occupied by another cell) and interactions with its neighbours. At the macroscopic level for simple contact interactions between agents and immediate neighbours, averaging this ‘microscopic’ level description gives rise to a non-linear diffusion equation [35], [36]. When introducing more general interactions between agents, aggregation patterns can be seen [37]. The averaged model, however, fails to reproduce cellular patterning.

A computational model (the cellular Potts or Glazier-Graner-Hogeweg model) for segregation between two cell populations, developed by Glazier et al. [38], explored use of cell surface energy as a function of its interactions with neighbouring cells and area constraints, to predict a configuration which minimises the energy of the entire system. The authors found that different surface energies, used to represent a range of cell-cell interactions, give rise to different levels of cell sorting or patterning. The notion where a cell displays preference to bind to one type of cell over others is known as “differential adhesion” and has been explored by various computational models and laboratory experiments (reviewed by [39], [28]). The cellular Potts model has been used to explore the rate of cell sorting due to differential adhesion associated with continuous and discrete variation in cadherin adhesion protein expression [30]. It was also employed to model cell positioning and segregation along the intestinal crypt epithelium, controlled by differential adhesion due to varying ephrinB/EphB activation, where the differential adhesion regulated the coordinated migration of cells within the crypt [40].

The varying adhesive and repulsive forces between different cell populations, which can result from Eph/ephrin interactions, have also been modelled by representing cells as spheres, which can attract or repel each other, giving rise to empirically observed cell sorting patterns: for example, attraction between like cells and repulsion between non-like cells, leading to distinct homoclusters [29]. A later approach, which simulates cell adhesion, de-adhesion and migration in greater detail [41], was used to model the time course of segregation of cells differing in cadherin expression. The results from this model were within 50% of the empirical data, but were less accurate when simulating the significantly faster rate of Eph-ephrin mediated cell segregation [42]. While faster cell segregation rates can be achieved by assuming the tendency of cells to follow their neighbours [26], such behaviour is not characteristic of Eph-ephrin signalling.

In this report we describe a mathematical approach that simulates Eph/ephrin- regulated cell-cell segregation and tissue boundary formation. Our model is based on first principles: empirical values for cell movement and cell proliferation and the assumption that Eph/ephrin signalling provides the dynamic control of adhesive and de-adhesive cell-cell interactions. We developed our model on the premise of condensing the multitude of molecular mechanisms and signal outputs that may contribute to cell-cell segregation into a small number of representative functions. We further restricted our model to the short time frame in which segregation of two cell populations takes place and consequently the loss of cells due to death is negligible. Accordingly, we suggest that tuneable terms describing (1) independent random cell motion on a substratum, (2) the sum of all possible Eph/ephrin-dependent adhesive and repulsive interactions between neighbouring cells, and (3) the rate of cell division, are sufficient to allow modeling of Eph/ephrin-controlled segregation of two cell populations. We aimed to accommodate the increasing number of protein interactions and signalling functions that are implicated in Eph-mediated cell positioning [1], [5], [6], [43]. We show that the model captures the dynamics of Eph/ephrin-mediated cell cluster formation and cell segregation, and predicts a range of outcomes that were found experimentally when the Eph/ephrin expression or activity levels were modulated.

## Results

### Mathematical Model

We and others [18], [19], [44] have previously used a two dimensional co-culture of ephrin-B1 and EphB2-expressing HEK293 cells to model Eph/ephrin mediated segregation of two cell populations. From these studies and based on existing knowledge of Eph/ephrin- mediated cell interactions, where active bi-directional signalling in Eph- and ephrin-expressing cells leads to cell-cell retraction and detachment of adhering cells, we derived the basic assumptions essential for simulating segregation between two distinct cell populations (see Methods S1, ‘Model Formulation’ for the detailed derivation and formulation of these assumptions). Our model thus includes terms describing (1) random cell motion on a substratum, (2) the distribution of adhesive and repulsive interactions between neighbouring cells and (3) the effect of cell division on the size of both cell populations. Our approach is designed to simulate Eph/ephrin-mediated cell sorting with a minimal number of empirical terms that are amenable to experimental validation and which are tuneable to match experimental observations.

Our model has two basic components: A stochastic differential equation (SDE) that describes the propagation in time of the position of each cell centre (terms 1 and 2), and a second component that controls the size of the cell population (term 3). In this experimental system, the rate of cell death is insignificant within the relevant time scales, so it is only necessary to subject each population size to a pure birth process: At a given time, 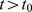, each existing cell can give birth to a new cell of the same type with the probability: 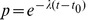
(1)


Where 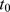 is the last time at which that cell divided (or was first created) and 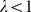 is the birth rate. The position of the centre of each cell is described by a stochastic equation of motion: The random term accounts for independent cell motion due to interactions with the substrate (term 1) and the deterministic term represents all interactions with other cells (term 2). The movement of the *i*'th cell centre is described by the SDE: 
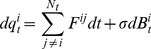
(2)


Where 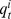 is the location of the centre of the *i*'th cell at time *t*, 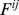 is the force exerted on the *i*'th cell by the *j*'th cell and 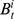 is an independent Brownian Motion. Equation 2 is derived from the equation of motion (see Methods S1, equation 2), assuming the drag force exerted by the substrate is large and the displacement due to internally generated cell motion is random with a normal distribution at the relevant time and length scales [31].

The interactions between cells are represented by radial forces (ie. dependent only on the distance between cell centres) and divided into two groups: attraction and repulsion forces **f**_r_ and **f**_a_ respectively. Thus 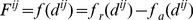
(3)with 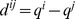, the distance between two cells. Further we take **f** to be a smooth function, so there exists a potential function _µ_, so that 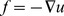.

We chose a particular functional form for the potential to represent cell surface tension and cell-cell adhesion: 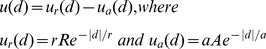
(4)


*R* and *A* set the magnitudes, and *r* and *a* set the length scales of the repulsion and attraction forces respectively. Subsequently 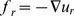, and 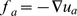.

The amplitudes and length scales are chosen to match the experimental system, and give an effective cell radius of 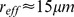 (see Methods S1 ‘Model formulation – Interaction forces’, for further details).

In the graphic representation of the simulation results, cells are plotted as circles of effective radius around the calculated position of their centres. Our model does not account for intermittent changes of cell size (e.g., during cell division), as the frequencies of these changes are rapid in comparison to the time scales of the experiments. Instead we assume a defined spatial range in which the attraction and repulsion forces between adjacent cells are effective and thus facilitate a well defined yet fluctuating cell size. The circles plotted are of the effective radius mentioned above and are to be seen as simplified representations of the real cells, of which the size and shape are changing.

When Eph-ephrin interactions take place, an additional term is introduced to attenuate the attraction due to cell-cell adhesion. The attraction term is countered by the function 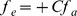, where 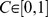 is an attenuation constant which controls the attenuation level of the attraction force. Thus, the total interaction between *i* and *j* cells is described by: 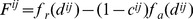
(5)with 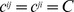 if *i* and *j* are cells of different types (i.e., Eph^+^ or ephrin^+^), and 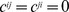 if the cells are of the same type (i.e. no Eph-ephrin interaction).

The corresponding biological situation occurs when an Eph containing cell meets an ephrin-containing cell and the adhesion force between the two cells is diminished, maximally to zero (when *C = *1). At this stage only a repulsion force due to surface tension, that does not allow cells to occupy the same space, will remain. This concept indeed gives rise to a form of ‘differential adhesion’, which unlike other models, also takes possible changes of the effective cell size into account (i.e. two adhesive cells will normally be closer together than two cells which have just detached due to the Eph-ephrin-facilitated cell-cell segregation).

### Comparison of simulated and experimental cell-cell segregation

#### Dynamics of cluster formation

**Figure 1:**
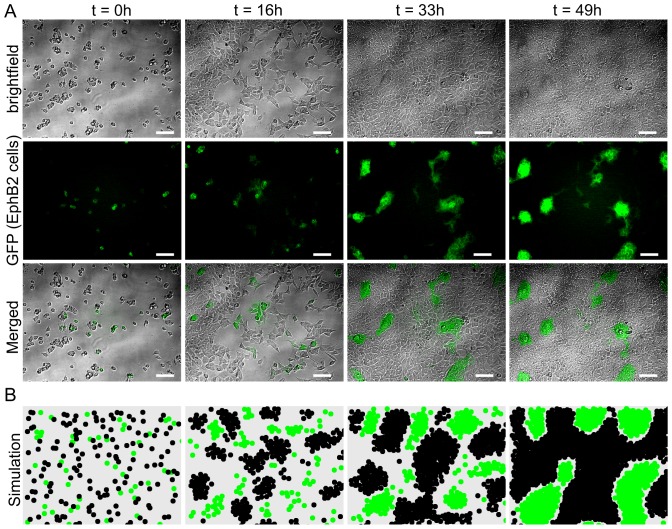
Time-lapse imaging and simulation of Eph/ephrin-driven cell-cell segregation. **A**) Representative time-lapse microscopic images (taken every 20 minutes for indicated times) from co-cultured EphB2/GFP (green) and ephrin-B1 (unstained) expressing HEK293T cells. Bright-field micrographs (top panels), green-fluorescent images (middle panels) and merged images (bottom panels) are shown, scale bars, 100 µm). **B**) Simulation of the same experiment; the corresponding time points are shown, ephrin-B1 and EphB2/GFP-expressing cells are represented as black and green circles, respectively.

We commenced the evaluation of our mathematical model, initially by comparing simulated and experimental cell-cell segregation in co-cultures of two HEK293 cell types, either co-expressing EphB2 together with membrane-targeted GFP (green fluorescent protein) for ease of visualisation [19], [44], or expressing the EphB2 ligand ephrin-B1. As previously reported [18], [44], using time-lapse fluorescence microscopy to record the continuous changes in the distribution and size of cell clusters, it was clear that within 49 hours, an initially mixed cell population, consisting of a 1∶3 ratio of GFP/EphB2 expressing (EphB2^+^) and ephrin-B1-expressing cells (ephrinB1^+^), gives rise to defined clusters of GFP-fluorescent EphB2-expressing cells that are surrounded by non-stained ephrin-B1 cells (Figure 1A, Video S1). We simulated this experiment, using the empirically-derived average speed of cell movement, cell proliferation rate and an initial Eph: ephrin cell ratio of 1∶3, adjusting the parameter values (see Methods S1 ‘Model formulation’) to: *A_eph_*  =  *A_ephrin_*  = 100, *R_eph_*  =  *R_ephrin_*  = 250, a = 7.5, r = 5.8 and 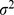  = 10.0, so that both cell populations would be exposed to equal inter-cellular adhesion and surface tension forces. Numerical solutions of the simulation, generating cell positions and graphic representations at defined time points, revealed that the relative size and distribution of the cell clusters were very similar in the simulated and experimental images through most of the time course of the experiment (Figure 1B, Video S2).

**Figure 2:**
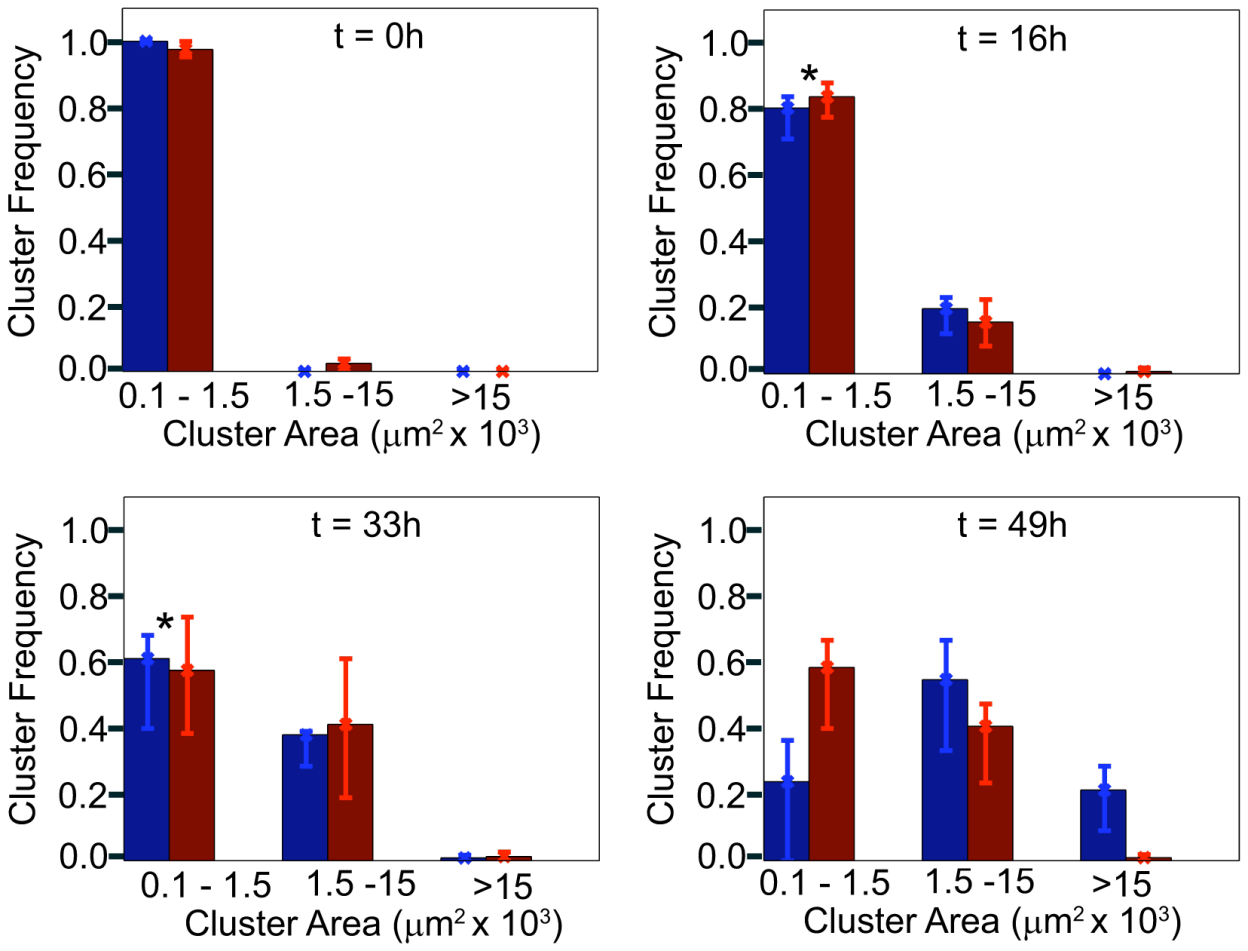
Distribution of cell cluster sizes during segregation. Clusters of EphB2/GFP expressing cells from the time course experiment in Figure 1 were grouped according to their footprint into three groups as indicated; bar graphs illustrate frequency of clusters in each bin at indicated time points, (red bars), experimental microscopy data; (blue bars), simulated data. Error bars represent the upper and lower bounds, below which 75% and 25% of the data points are included. *Multivariate-ANOVA, p>0.5.

To compare quantitatively the simulated and experimental cell-cell segregation, we analysed images from 6 experiments and a corresponding set of 6 independent simulations/numeric realisations. While direct comparison of experimental and simulated clusters suggested very similar size distributions, for ease of comparison, cell clusters (green) were sorted by their areas into three groups: small (100–1500 µm^2^), medium (1500–15000 µm^2^) and large (>15000 µm^2^) cell clusters. The group boundaries were chosen, as described previously [18], to reflect closely the actual cell patterning behaviour, thereby capturing the majority of all observed clusters in the small- and medium-size cluster bins, while less frequent large clusters that formed mainly at the end of the simulation were captured in a third bin (Figure 2).

Multivariate-ANOVA analysis of the cluster distributions suggested very similar frequencies of small and medium-sized clusters forming in the experiment and the simulation up to the 33 hour time point (p = 0.5317). However, by the final time point (49h) there were significant differences (p = 0.0028) between the simulated and experimental data. The increased number of small (100–1500 µm^2^) clusters in the experiment as compared to the simulation is due to some single cells remaining separated from the larger clusters when increasing cell density prevents their effective sorting so they therefore remain in ‘small’ clusters. This apparent variability in the cell population (i.e. the occurrence of ‘slow cells’) may be caused by variability in Eph/ephrin surface expression levels between individual cells, which is not present in the simulated cell population. Similarly, the significant number of large simulated clusters (>15000) at the 49 h time point was not reflected by the experiment, again likely to be partly due to inhibited sorting at higher cell density. In addition, detailed analysis of the corresponding microscopic images (Figure 1A) revealed that the very tight compaction of EphB2-positive cells reduced the overall footprint of clusters and of individual cells at the end of the experiment. By contrast, the simulation assumes equal adhesion and repulsion forces within both cell populations throughout the experiment, so that simulated clusters appear larger than the experimentally observed clusters. This suggests that during Eph/ephrin-regulated cell sorting, clustering of the EphB2 cell population may be modulated according to the expression level and kinase activity of the Eph receptors [1], [5].

#### Modulation of the Eph-ephrin signalling strength effects cluster formation

We assessed the relationship between the Eph-ephrin signal strength and cluster size distribution on the basis of previously-published co-culture experiments, where the effects of modulating expression level and kinase signalling activity of Eph receptors on cell segregation and cluster formation had been analysed [44]. That study revealed that changes in the composition of Eph signalling complexes, by additional exogenous expression and recruitment of kinase-active or kinase-inactive receptors, can either increase cell contraction and clustering or promote spreading and intermingling, respectively [44]. To accommodate the complexity of Eph cell signalling, we modulated the attenuation constant *C* (see equation 5) to simulate the uneven repulsion and/or attraction forces within the two different populations (*R_eph_ ≠ R_ephrin_* and *A_eph_ ≠ A_ephrin,_* respectively).

**Figure 3:**
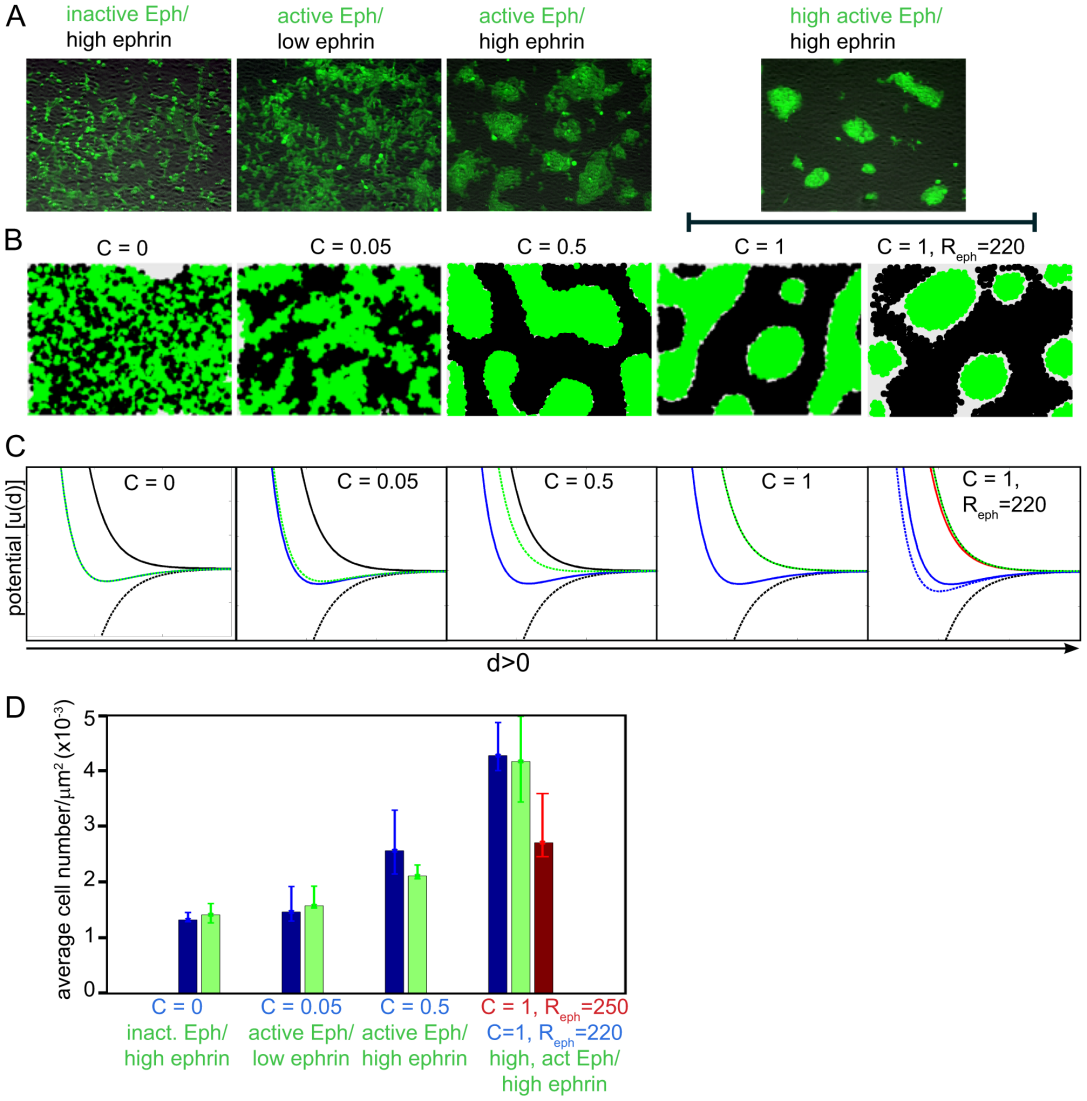
Eph expression level and signalling capacity regulate cell segregation: comparison of experimental versus modeling outcomes. **A**) Increased Eph signalling capacity results in enhanced cell-cell segregation [44]: HEK293 cells expressing cytoplasmic deleted (signalling-inactive), wild type EphB2 or co-expressing EphB2 and EphA3 were co-cultured with cells expressing low or high levels of ephrin-B1, as indicated. EphB2 cells were stained with Cell-Tracker green for ease of visualisation, images taken after 48 h co-culture when cell-cell segregation was regarded as complete. **B**) Simulation of the same experimental conditions, using parameter values of: *A_eph_  =  A_ephrin_  = *100, *R_eph_  =  R_ephrin_  = *250, *a* = 7.5, *r* = 5.8 and 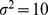, apart from the right-most panel, where *R_eph_  = 220.* The initial ratio of Eph: ephrin cells in all cases is 1∶1. **C**) Functions of the force potential, u(d) between two cells at distance d>0, for the simulations illustrated in B. Unbroken black line,u_r_,is the potential of the repulsion force at *R_eph_  = 250*; Unbroken red line,u′_r_,is the potential of the repulsion force at *R_eph_  = 220*; Broken black line, u_a_, is the potential of the attraction force; Unbroken blue line,u_r_ − u_a_,is the potential of the total force between same type cells *R_eph_  = 250*; Broken blue line,u′_r_ − u_a_,is the potential of the total force between same type cells when *R_eph_  = 220*; Broken green line, u_r_ − (1−C)u_a_, is the potential of the total force between cells of different types; **D**) Statistical analysis of cluster characteristics from a minimum of 5 independent data sets: Comparison of the cellular densities that were observed experimentally by microscopy (green bars), or by simulation under the conditions detailed in panels C (blue bars; except for *C* = 1, *R_eph_*  = 250, red bar). For microscopic images the number of cells in a cluster was estimated from the total fluoresence intensity of the cluster, divided by the average fluorescence intensity of a single cell as detailed previously [44]. Error bars represent the upper and lower bounds, below which 75% and 25% of the data points are included.

In the experiments, over-expression of the inactive EphB2 mutant lacking the intracellular domain (Figure 3A, left column) completely blocks cell-cell segregation and cluster formation, compared to Wt EphB2 expressing cells (Figure 3A, third column), when co-cultured with cells expressing ephrinB1. In comparison, co-cultures of Wt EphB2-expressing cells with parental HEK293 cells, which express low but detectable levels of ephrin-Bs (Figure S1), results in an intermediate phenotype, revealing some segregation between the two cells types, but lacking apparent clusters or clear cluster boundaries (Figure 3A, 2^nd^ column). Modulation of the attenuation constant of the attraction force *C* allowed us to simulate these conditions, so that increasing the values of *C* from 0 to 1 effectively introduced increasing de-adhesion/segregation between the simulated Eph and ephrin cell populations (Figure 3B,C).

**Figure 4:**
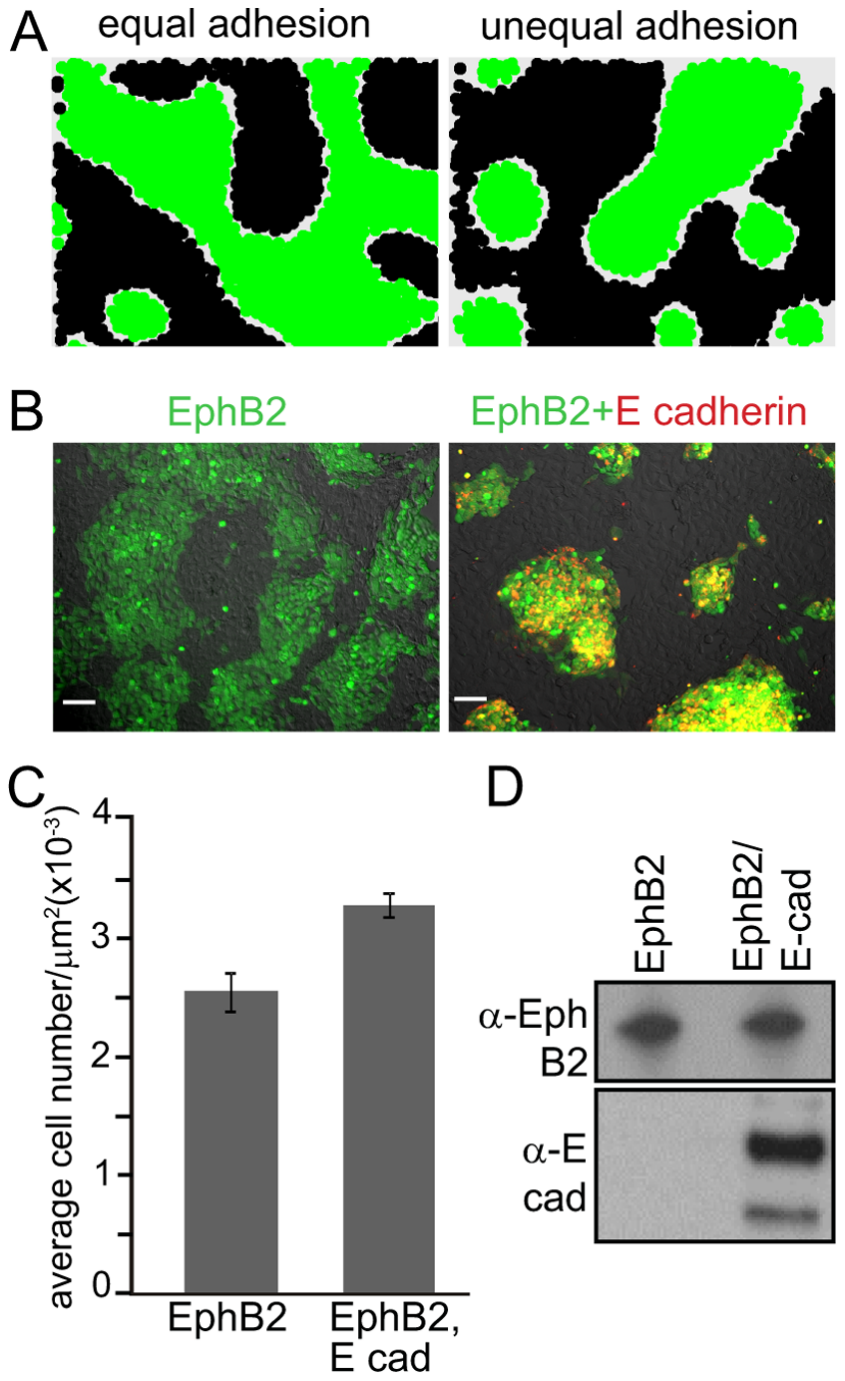
Increased cell-cell adhesion within one cell population is required for the formation of tightly packed cell clusters. **A**) Simulation of cell-cell segregation using the same adhesion term in both cell types (*A_eph_*  =  *A_ephrin_*  = 100, left panel) vs. increased adhesion only in the green cell population (*A_eph_*  = 110, *A_ephrin_*  = 100, right panel). Both simulations started with the same number of Eph (green) and ephrin (black) expressing cells. In the “Equal adhesion” case, an ‘Islands-in-a-sea’ pattern is less apparent. **B**) Representative images from segregation assays of unlabelled ephrin-B1 cells co-cultured with Cell Tracker-green labelled (green staining) EphB2 cells, without (left) or with E-cadherin-cherry expression (red staining, right); scale bar, 75 µm. **C**) Quantitation of cell densities in the cell clusters shown in B (n = 10). **D**) Western blot analysis of lysates from parental and E-cadherin-cherry-transduced cells, using the indicated antibodies.

We examined an extreme case of clustering, where over-expression of a second signalling-competent Eph receptor, EphA3, increases the cellular response in EphB2-expressing cells to the interaction with ephrin-B1 cells and results in enhanced cell segregation [44]. In this experimental setting, the clusters of Eph-expressing cells appear brighter, as the cells condense and become more tightly packed than the corresponding ephrin cells, thereby effectively decreasing the footprint of individual clusters (Figure 3A, right-most panel). In this case, even a maximal attenuation constant of *C* = 1 was inadequate for simulating the corresponding increase in the packing of individual EphB2 cells within clusters (Figure 3B). Therefore we considered that modeling of the increased cell compaction within the EphB2/EphA3 clusters might require lowering the repulsion term, or alternatively increasing the attraction force within the Eph-expressing cell population (see Figure 4 below), to effectively increase cell packing beyond that seen in the ephrin-expressing cells. By reducing the repulsion strength, which simulates cell surface tension (see equation 4), of EphB2 cells to *R_eph_*  = 220 *≠ R_ephrin_*  = 250, and thereby decreasing the effective radius of Eph expressing cells relative to that of ephrin expressing cells, the simulation now yielded a pattern of tightly-clustered Eph cells in a “sea” of ephrin-expressing cells that closely matched the experimentally-derived pattern (Figure 3B, right-most panel). Note, this decrease in the effective radius of Eph cells makes them pack more closer together, and therefore leaves more space for the ephrin cells, evident by the space left in the simulation even after 48 hours, whereas in the laboratory experiment, the ephrin cells expand to occupy the free space.

Quantitation and statistical (ANOVA) analysis of the experimental and simulated cell densities in the Eph cell clusters (Figure 3D), suggested a close fit between experimental and simulated data (p = 0.506), whereby in particular, adjustment of the cell density by decreasing the cell surface tension of the EphB2/EphA3-expressing cells resulted in simulated cell densities which closely matched the experimental data. Our observations suggest that the cell morphology and cell-cell adhesion within these clustered Eph-expressing cell populations are changed compared to the surrounding ephrin-expressing cells, in agreement with a range of reports on mechanisms of Eph/ephrin-facilitated tissue patterning and boundary formation [23], possibly due to Eph receptor crosstalk with cell-cell adhesion molecules [45].

#### The formation of tightly-packed “Islands-in-the-sea” Eph cell clusters relies on enhanced cell-cell adhesion within clusters

We went on to examine crosstalk between Eph signalling and cell-cell adhesion proteins, by simulating the effect of altering the relative adhesion within one of the two cell populations. As a starting point we considered two equally sized populations of Eph and ephrin expressing cells with identical adhesion forces between the two distinct cell types (*A_eph_  =  A_ephrin_*). While in this setting of the simulation, the two cell populations clearly segregated from each other, a preference for clustering of one cell type over the other was not apparent and the segregation did not yield the typical ‘Islands-in-a-sea’ pattern observed in other experiments (Figure 4A, left panel). However, increasing the cell-cell adhesion term in one of the simulated cell populations over the other (*A_eph_ ≠ A_ephrin_*), resulted in tighter clusters of this population and led to islands within the sea of cells of the other type (Fig 4A, right panel). We tested this prediction experimentally: cell-cell adhesion was increased within the EphB2-expressing cell population by overexpressing the cell-cell adhesion protein E-cadherin. This was achieved by lentiviral transduction of mCherry-tagged E-cadherin [45] into the EphB2-expressing HEK293 cells, which lack endogenous E-cadherin, but in agreement with their neural origin [46] express N-cadherin. As illustrated in Figure 4B,C, overexpression of E-cadherin indeed resulted in the ‘islands in a sea’ pattern predicted from the simulation, due to the formation of distinct EphB2 cell clusters with significantly enhanced cell density as compared to the parental cells which lack E-cadherin. Western blotting confirmed the expression of the mCherry-E-cadherin in the transduced cells, and that the transduced and control cells maintained similar levels of EphB2 (Fig 4D).

Our results indicate that our mathematical model, based on the concept that the sum of various Eph and ephrin-derived signal inputs is translated into cellular responses which can be represented by adjustable terms for cell-cell adhesion and repulsion, provides the robustness and flexibility to allow realistic simulations of Eph/ephrin-regulated cell sorting under a range of experimental conditions.

## Discussion

Eph-ephrin interactions between neighbouring cells control the dynamic changes in cell morphology, adhesiveness and motility that underlie tissue patterning and boundary formation. As the number of cell-biochemical pathways implicated in these processes grows, the difficulty in predicting outcomes from knowledge of individual signalling pathways increases [17]. It is clear that prediction of *in vivo* and *in vitro* cellular behaviour during patterning can be assessed by mathematical modeling and several approaches have been used to aid our understanding of molecular and cellular concepts underlying cell positioning and tissue patterning [7], [22]–[24].

In this manuscript we described a mathematical approach to simulate modes of Eph/ephrin-controlled 2-dimensional cell-cell segregation and aggregation. On the basis of a stochastic Lagrangian process and by the incorporation of empirical parameters for cell movement and cell proliferation, we developed a mathematical model that allows both a realistic simulation of Eph/ephrin-directed segregation between two cell populations and the modelling of responses to modulation of adhesive and repulsive signalling mechanisms. Direct comparison of experimental and computer-simulated formation of cell patterns shows that our model recapitulates the spatial and temporal dynamics of the cell-cell segregation processes.

The cellular Potts model, a computational model based on the concept that differences in binding energies between two interacting cell populations direct the separation between non-like cells (the differential adhesion hypothesis), has been adopted to simulate EphB/ephrin-B controlled cell sorting along the intestinal crypt epithelium [40]. An alternative approach, including a term for cell density-dependent cell motility [41], was used to model segregation in 2-D tissue culture between two cell populations that differ in their expression of the cell-cell adhesion protein E-cadherin. However, in the case of Eph/ephrin regulated cell-cell interactions, the kinetics of the simulated sorting lagged behind the tissue culture experiment by an order-of-magnitude [42], suggesting that additional terms may be required to model sorting between Eph- and ephrin-expressing cells.

Considering the characteristic dichotomous functions of Eph receptors, eliciting either cell-cell repulsion and segregation or cell-cell adhesion [1], [5], we developed a model for the behaviour of two intermingled cell populations that display differing adhesive/repulsive forces within and between each population [29]. We took a novel approach of complementing terms of surface tension (representing cell-cell repulsion) and attraction (representing cell-cell adhesion) that can be adjusted according to the Eph/ephrin signal strength. We are aware that on a molecular level Eph/ephrin-facilitated cellular patterning relies on a fine-tuned interplay of a large number of known and potentially an even larger number of unknown signalling pathways [17], however, we propose that collapsing this complexity to a selected, small number of measurable mathematical terms, which can approximate the sum of all contributions, provides a model that realistically reflects the overall processes. Thus, we introduce a Brownian motion term to represent the movement of a single cell that is not influenced by any neighbours and bundle all possible interactions between two cells into two radial forces: a repulsion term that represents the sum of cell surface tension and spatial exclusion forces that prevent cells to occupy the same space, as well as an attraction term which represent all adhesion forces, including those governed by cadherin interactions. For any cell type, the size of parameters controlling the Brownian motion and the strength and length scale of both radial forces can be estimated directly from the paths of cells moving independently and at a density where interactions with neighbouring cells occur (Aharon et al, manuscript in preparation). In this model, the Eph-ephrin regulated interaction can act to cancel part or all of the adhesion forces between Eph and ephrin expressing cells. The characteristics of these forces impose a stronger (spatial) separation between the two different types of cells. This heterologous interaction promotes the retraction of Eph- and ephrin- expressing cells from each other, as well as diminishing the adhesion between them.

Computer simulations, based on numerical solutions of our model, allowed the prediction of the dynamics of cell segregation and cluster formation. A comparison to experimental data demonstrated our model's capacity to reproduce the spatial and temporal dynamics of cluster formation seen in the tissue culture experiments. In contrast to previous models of Eph/ephrin-regulated cell segregation, our model adjusts for the proliferative increase in number of cells in the two different cell populations throughout the experiment. As cell density increases, cells in our model are inherently slowed down due to interactions with increasing numbers of neighbouring cells. We find that cell proliferation is an important contributor to the dynamics of cluster formation.

We demonstrate the capacity of our model to accommodate variation in Eph expression and strength of signalling function, by modulating the amplitude of those model parameters that control the strength of the Eph-ephrin interaction (Figure 3). A comparison between our model-based predictions and experimental cell behaviour revealed the necessity to include asymmetry in the adhesive behaviour of the Eph and ephrin-expressing cell population in order to arrive at the typical cellular pattern, where one population of tightly-packed cells with increased adhesion (or accordingly-reduced repulsion) between like cells is surrounded by a “sea” of less-tightly packed cells of the other type (Figure 4). Specifically, our model highlighted that ephrin/Eph-driven cell retraction/repulsion at the interface between the two cell populations is insufficient to generate this typical “Island-in-the-sea” pattern and pointed to the critical role of additional cell-cell adhesion within the Eph-expressing population that is required to generate the tighter cell packing in this population. Interestingly, this same conclusion was previously drawn from experiments addressing EphA4/ephrin-B2-driven rhombomere formation in Zebrafish, where EphA4-expressing cells show increased adhesion to each other [47]. Since the cell-cell adhesion protein E-cadherin is known for its co-ordinated and co-localised expression with Ephs at sites of cell-cell contacts [48] and is implicated in Eph/ephrin-facilitated boundary formation [45], we over-expressed mCherry-E-cadherin in EphB2 cells. Our experiments confirmed a role for unequal adhesion in generating the asymmetry between the enclosed (i.e., the “island”) and the enclosing (i.e., the “sea”) cell populations. Eph/ephrin and E-cadherin interactions control the partitioning of two cell populations, by co-ordinately controlling the cell segregation between, and the adhesion within, each population.

In summary, we conclude that a simple model based on terms defining differential cell-cell adhesion, in which cell-cell adhesion forces are balanced to varying degrees by Eph-ephrin regulated cell-cell de-adhesion, is sufficient to describe the cell patterning observed between Eph and ephrin expressing cell populations migrating on a 2-dimensional surface over time.

## Methods

### Experimental setup

Segregation assays of EphB2- and ephrin-B1-expressing HEK293 cells were performed as described previously [44], using approx. 50,000 cells seeded into a well (0.8 cm^2^ surface area) of a cell culture slide pre-coated with 10 µg/ml fibronectin. To monitor cell movement over the course of the experiment (48–60 h) we used time-lapse fluorescent microscopy (Leica AF6000LX microscope), taking images every 20 minutes. For experiments in Figure 3 and 4, images were taken at the end of the experiment when cells achieved confluence. In Figure 3, images are shown from previously reported experiments [44] using EphB2 cells stably transfected with the indicated constructs and labelled with Cell Tracker Green (Invitrogen). For experiments in Figure 4, EphB2 cells expressing E cadherin-cherry (kindly provided by E. Batlle) were produced by lentiviral transduction and sorted by flow cytometry. Western blots of cell protein extracts were with antibodies against EphB2 (R&D Systems) and E-cadherin (Abcam). Immunoprecipitation and Western blot of ephrin-Bs (Figure S1) was with anti-ephrin-B1/2/3 antibody C-18 (Santa Cruz).

### Numeric solutions

A JAVA program was written to solve a discretised version of the model's equations, and obtain


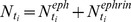
 and 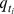 (using a 1st order Euler method), for 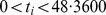 [sec], given all other model parameters as inputs.

Model parameters set directly from observations of their physical sizes, are: 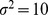 [µm^2^/min], 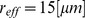 and 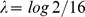 [1/h]. Other parameter values were: *A_eph_*  =  *A_ephrin_*  = 100, *R_eph_*  =  *R_ephrin_*  = 250, *a* = 7.5 and *r* = 5.8.

Output was generated as images in which each cell is represented as a circle of radius r_eff_ around its calculated position and colour coded according to its cell's type. A video file, made of all images, and a.csv file that includes the position of all cells at all calculated times were also generated.

### Image analysis

Analysis of both simulation and experimental results was done using ImageJ together with a designated MATLAB code, generated using the ImageJ analysis package. For the analysis of microscopic images, thresholds were adjusted to guarantee optimal performance in capturing cell clusters with the least possible noise. Once clusters were identified, their areas and fluorescence intensities were recorded. The number of cells in a cluster was then estimated from the intensity by dividing by the average intensity of single cells determined from the images.

Images obtained from simulations have only three set colours (green, black and white) and therefore the threshold for identifying Eph (i.e. green) clusters is the same for all simulated setups. Once the clusters were identified, areas were recorded. The number of cells within each cluster was then obtained by crossing the data regarding all clusters positions and the.csv file containing all cell positions at the relevant time.

## Supporting Information

Figure S1 Ephrin-B levels in model cell lines. Anti-ephrin-B1/2/3 immunoprecipitates from lysates of ephrin-B1-transfected or parental HEK293 cells (equalised for total protein content) were analysed by Western blot with anti-ephrin-B1/2/3 antibody (top panel). Equal loading was verified by anti-GAPDH Western blot of total cell lysates (bottom panel).
(TIF)

Methods S1 Model Formulation: Derivation and formulation of assumptions on cell proliferation, individual cell motion, and interaction forces between cells.
(DOC)

Video S1 Time-lapse microscopy of co-cultured HEK293 cells co-expressing EphB2 together with membrane-targeted GFP and cells expressing the EphB2 ligand ephrin-B1. The changes in the distribution and size of cell clusters were recorded over 49 hours at 1 frame/20 min. The movie is displayed at 3 frames/second.
(MP4)

Video S2A simulation of the time course of cell-cell segregation illustrated in video 1, using the empirically-derived parameters for cell movement, cell proliferation rate and the initial Eph: ephrin cell ratio of 1∶3, with parameter set at: A_eph_  =  A_ephrin_  = 100, R_eph_  =  R_ephrin_  = 250, a = 7.5, r = 5.8 and 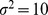 (equal adhesion and surface tension forces).(MP4)
