# Supplementary figures and images for: A Mathematical Model for Eph/Ephrin-Directed Segregation of Intermingled Cells
Source: PLoS One. 2014 Dec 1;9(12):e111803. doi: 10.1371/journal.pone.0111803 (PMC4249859; doi:10.1371/journal.pone.0111803)

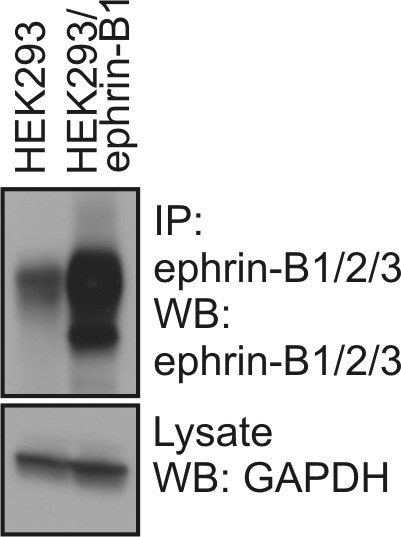

Supplement: Figure S1 — Ephrin-B levels in model cell lines. Anti-ephrin-B1/2/3 immunoprecipitates from lysates of ephrin-B1-transfected or parental HEK293 cells (equalised for total protein content) were analysed by Western blot with anti-ephrin-B1/2/3 antibody (top panel). Equal loading was verified by anti-GAPDH Western blot of total cell lysates (bottom panel). (TIF) [file pone.0111803.s001.tif]
