# Supplementary material for: A Mathematical Model for Eph/Ephrin-Directed Segregation of Intermingled Cells
Source: PLoS One. 2014 Dec 1;9(12):e111803. doi: 10.1371/journal.pone.0111803 (PMC4249859; doi:10.1371/journal.pone.0111803)
Supplement: Methods S1 — Model Formulation: Derivation and formulation of assumptions on cell proliferation, individual cell motion, and interaction forces between cells. (DOC) [file pone.0111803.s002.doc]

**Methods S1- Model Formulation**

**Model variables**

$N_{t}$ Number of cells at time *t*

$N_{t}^{eph}$ Number of Eph expressing cells at time *t*

$N_{t}^{ephrin}$ Number of ephrin expressing cells at time *t*

$\mathbf{q}_{t}$ Positions of all cells at time *t*

$\mathbf{q}_{t}^{i}$ Position of the i’th cell at time *t*

**Empirical Constants**

$\sigma$ ‘Free’ diffusion rate

$\lambda$ Proliferation rate

$a$ Attraction length scale

$r$ Repulsion length scale

$A$ Attraction magnitude

$R$ Repulsion magnitude

Empirical constants set directly from observations of their physical sizes, are: $\sigma^{2}=10\text{ [}\mu\text{m}^{2}/\min]$ and $\lambda=\log2/16$ [1/h]. Other parameters (*A, R a* and *r*) are constrained by the size of a real cell: First to insure forces do not extend too far beyond the cell’s surface, and further to insure the equilibrium distance ($r_{eff}$, definition below) matches the radius of a single cell, which we take to be $15\text{ }\mu\text{m}$ based on the radius of an average cell in the experimental system.

In following work we demonstrate how *A,R,a,* and *r* can be estimated from data regarding cell paths, using Maximum-Likelihood Estimation (Aharon et al, in prep).

**Scope of the Model**

In our laboratory experiments we model two cell populations stably expressing either EphB2 or ephrin-B1 that were co-cultured within an area of approximately $500\times700\text{ }\mu\text{m}$ (representing the microscope field of view). The EphB2 population was identified with co-expressed membrane targeted green fluorescent protein (GFP), or by labelling with Cell Tracker green. The cell seeding density in the culture dish at the start of the experiment was 50,000 cells/0.8 cm^2^, corresponding to 220 cells/field of view, whereby their proliferation rate was estimated from their doubling every 16 hours. Based on the length and temporal scales of interest, and the fact these are much larger than those of a single cell, we set our model to describe the position of each individual cell, but without considering intracellular interactions.

Accounting for each individual cell allows a smooth transition from very low to very high cell density. The initial number of cells in the simulation is taken to match the experimental initial conditions, and after this, each cell can ‘give birth’ to a daughter cell, following a Poisson process of rate $\lambda$. The daughter cell will always be of the same type (i.e. Eph or ephrin expressing) as the parent.

***Assumption* 1*: Cell proliferation.*** *Assume each population size follows a pure birth process. At a given time* $t>t_{0}$*, each existing cell can give birth to a new cell of the same type with probability* $p=e^{-\lambda(t-t_{0})}$*, where t_0_ is the last time at which that cell divided (or was first created).*

At any given time *t*, the total number of cells $N_{t}$ is a random variable. The expected value of the total number of cells at time *t* is:

$\text{E}N_{t}=N_{0}e^{\lambda t}$ (1)

Since the cell population size doubles every 16 hours, we set: $\lambda=\log2/16$.

The position of the centre of each cell is described by an equation of motion, which includes a random term to account for internally generated cell motion, and a deterministic representation of all external effects. For the *i*’th cell, let $\mathbf{F}^{ij}$ be the force due the interaction with cell *j*. Assume the drag force due to interaction with the substrate $-\delta V^{i}$ is large so $m<<\delta$ and $1/\delta\approx O(1)$, where $\delta$ is the drag coefficient and $m$ is the mass of a single cell.

Then the equation of motion is

$\begin{matrix} md\mathbf{V}_{t}^{i}=\left(\sum_{j\neq i}^{N_{t}} \mathbf{F}^{ij}-\delta\mathbf{V}_{t}^{i}\right)dt+\tilde{\sigma}d\mathbf{W}_{t}^{i} \\ \mathbf{V}_{t}^{i}dt=\sum_{j\neq i}^{N_{t}} \mathbf{F}^{ij}dt+\sigma d\mathbf{W}_{t}^{i} \\ d\mathbf{q}_{t}^{i}=\sum_{j\neq i}^{N_{t}} \mathbf{F}^{ij}dt+\sigma d\mathbf{W}_{t}^{i} \end{matrix}$ (2)

Where $\mathbf{V}_{t}^{i}$ is the velocity of the centre of cell *i* at time *t*, $\mathbf{q}_{t}^{i}$ is its position, and $\mathbf{W}_{t}^{i}$ is some independent random term. $\sigma$ is a constant which determines the rate of independent motion.

At this scale, it has been shown that the Mean Square Displacement (MSD) of a single cell which is moving freely (without interactions with other cells) is linear in time , i.e. $\left\langle\left(\Delta\mathbf{q}^{i}\right)^{2}\right\rangle\propto\Delta t$. We therefore take $\mathbf{W}_{t}^{i}$ to be a Brownian motion: $\mathbf{B}_{t}^{i}$, and choose the value of $\sigma$ based on our experimental data: $\sigma^{2}=\frac{\left\langle\left(\Delta\mathbf{q}^{i}\right)^{2}\right\rangle}{\Delta t}\approx10\frac{[\mu m]^{2}}{[\min]}$. One can summarise this into a single assumption:

***Assumption* 2*: Individual cell motion.*** *The motion of a single cell can be described by a SDE of the form:* $d\mathbf{q}_{t}^{i}=\sum_{j\neq i}^{N_{t}} \mathbf{F}^{ij}dt+\sigma d\mathbf{B}_{t}^{i}$. *Where* $\mathbf{q}_{t}^{i}$ *is the location of the centre of the i'th cell at time t,* $\mathbf{F}^{ij}$ *is the force exerted on cell i due to the interaction with the j’th cell, and* $\mathbf{B}_{t}^{i}$ *is an independent Brownian Motion (all vectors are of order 2).*

**Interaction forces**

We assume that interactions between cells can be represented by radial forces, i.e. depend only on the distance between them, and can be classified into two groups: attraction and repulsion forces.

***Assumption* 3*: Radial force.*** *Assume the force* $\mathbf{F}^{ij}$ *between the i’th and j’th cells located at* **q***^i^ and* **q***^j^ respectively depends only on the distance between them:* $\mathbf{F}^{ij}=\mathbf{f}(\mathbf{q}^{i}-\mathbf{q}^{j})$*. If we further assume that* **f** *is a smooth function, then ∃ a potential function* *, s.t* $\mathbf{f}=-\nabla u$*.*

***Assumption* 4*: Repulsion and attraction forces.*** *For any pair of cells one can write:* **f** = **f***_r_* − **f***_a_. Where* **f***_r_ is the repulsion force and* **f***_a_ is the attraction force. These are characterised by their amplitudes R, A and their length scales r, a. We further assume* **f***_r_ and* **f***_a_ are positive, monotonically decreasing functions.*

Of note, these assumptions are often used in deterministic descriptions of swarm behaviour (see Mogilner et al (2003), for example). In our case the repulsion force can be identified with surface tension and the attraction force with adhesion. The amplitudes and length scales are chosen to match the biological system, and were found to agree with the rule given in Mogilner et al (2003) (strong short term repulsion and weak long term attraction), which ensures the existence of a stable aggregate in the case of the deterministic system. (i.e. for a single population type, when $\sigma=0$ and $N>>1$ there exists a locally stable solution with equal distances between agents).

The choice of parameter sizes within this region, also determines the equilibrium distance between two cells in the deterministic system ($\sigma=0$) of only two identical cells (*N* = 2). This distance will be referred to as the effective radius, *r_eff_*. Since in the real system all interactions actually take place at the cell surface, this radius is used to represent the effective radius of all cells. When presenting the model’s output graphically, we use this radius to determine the size of the circle we draw around the calculated position of each cell’s centre.

We set the value of *r_eff_* based on observations of the radius of an average cell. It does not have any actual role in the model but to impose a constraint on the relations between *A*, *R*, *a* and *r*. For the specific choice of potential function given in equation 8, a direct calculation gives $r_{eff}=\frac{ar\log(Ar/Ra)}{2(r-a)}$.

We further allow **f***_r_* and **f***_a_* to be different between the Eph and ephrin expressing cell populations by setting *A = A_eph_* and *R = R_eph_* to give $\mathbf{f}^{eph}$, or *A = A_ephrin_* and *R = R_ephrin_* to give $\mathbf{f}^{ephrin}$ for each subpopulation respectively.

**Eph-ephrin effect**

This assumption is made to capture the modulation of cell-cell interactions due to Eph-ephrin regulation, which come to play in interactions between the two different subpopulations (Eph and ephrin expressing cells). When an Eph receptor on a cell membrane binds with an ephrin ligand on an adjacent cell, active signalling promotes re-organisation of the cell cytoskeleton and ensuing cell rounding and proteolysis of interacting proteins, leading to a decrease in the adhesion between the two cells. This decrease in adhesion can be modulated based on the levels of Eph and ephrin expression within the cell membrane. To capture this we introduce an additional term: $\mathbf{f}_{e}=+C\mathbf{f}_{a}$, where $C\in[0,1]$ is a parameter that allows regulating the strength of the Eph-ephrin interruption to the normal adhesion between the different cells by choosing the value of .

***Assumption* 5*: The Eph-ephrin cell-cell segregation effect.*** *When Eph-ephrin interaction takes place, an additional repulsion term is introduced to attenuate the attraction due to adhesion. The attraction term is countered by* $\mathbf{f}_{e}=+C\mathbf{f}_{a}$*, where*  *is a constant which controls the attenuation*

*level of the attraction force. Now* $\mathbf{f}(\mathbf{q}^{i}-\mathbf{q}^{j})=\mathbf{f}_{r}(\mathbf{q}^{i}-\mathbf{q}^{j})-(1-c^{ij})\mathbf{f}_{a}(\mathbf{q}^{i}-\mathbf{q}^{j})$*, with* $c^{ij}=c^{ji}=0$ *if the cells are of the same type (i.e. no Eph-ephrin interaction), or* $c^{ij}=c^{ji}=C$ *if the i’th and j’th cells are of different types (i.e one is Eph and the other is an ephrin expressing cell).*

In the case where the forces between same type cells are different between the two populations, we set two other parameters: *A_eph:ephrin_* and *R_eph:ephrin_* to avoid ambiguity.

**A specific choice of potential function**

We introduce a specific choice for the potential function:

$u(\mathbf{d})=rRe^{-|\mathbf{d}|/r}-aAe^{-|\mathbf{d}|/a}+c^{ij}aAe^{-|\mathbf{d}|/a}$ (3)

The first term gives the repulsion force between cells and the second term gives the attraction force. The last term gives rise to the Eph-ephrin regulation: when *i* and *j* are of the same type, it equals zero, and when *i* and *j* are of different types, $c^{ij}=C$, so it attenuates the adhesion force.

More specifically, for cells *i* and *j* located at $\mathbf{q}^{i}=(q_{x}^{i},q_{y}^{i})$ and $\mathbf{q}^{j}=(q_{x}^{j},q_{y}^{j})$ respectively, we have $\mathbf{d}^{ij}=\mathbf{q}^{i}-\mathbf{q}^{j}$.

Thus:

$\begin{matrix} \mathbf{f}_{r}(\mathbf{d}^{ij})=Re^{-|\mathbf{d}^{ij}|/r}\left(\frac{q_{x}^{i}-q_{x}^{j}}{|\mathbf{d}^{ij}|}\cdot\hat{x}+\frac{q_{y}^{i}-q_{y}^{j}}{|\mathbf{d}^{ij}|}\cdot\hat{y}\right) \\ \mathbf{f}_{a}(\mathbf{d}^{ij})=Ae^{-|\mathbf{d}^{ij}|/a}\left(\frac{q_{x}^{i}-q_{x}^{j}}{|\mathbf{d}^{ij}|}\cdot\hat{x}+\frac{q_{y}^{i}-q_{y}^{j}}{|\mathbf{d}^{ij}|}\cdot\hat{y}\right) \\ \mathbf{f}_{e}(\mathbf{d}^{ij})=c^{ij}Ae^{-|\mathbf{d}^{ij}|/a}\left(\frac{q_{x}^{i}-q_{x}^{j}}{|\mathbf{d}^{ij}|}\cdot\hat{x}+\frac{q_{y}^{i}-q_{y}^{j}}{|\mathbf{d}^{ij}|}\cdot\hat{y}\right) \end{matrix}$ (4)

**Additional Reference**

Mogilner A, Edelstein-Keshet L, Bent L, Spiros A (2003) Mutual interactions, potentials, and individual distance in a social aggregation. Journal of Mathematical Biology 47: 353–389.
